# Yeast-based production and in situ purification of acetaldehyde

**DOI:** 10.1007/s00449-022-02697-w

**Published:** 2022-02-08

**Authors:** Hendrik G. Mengers, William Graf von Westarp, Daniela Brücker, Andreas Jupke, Lars M. Blank

**Affiliations:** 1 Institute of Applied Microbiology - iAMB, Aachener Biologie und Biotechnologie - ABBt, RWTH Aachen University, Aachen, Germany; 2 Fluid Process Engineering (AVT.FVT), RWTH Aachen University, Aachen, Germany

**Keywords:** Acetaldehyde, *S. cerevisiae*, In situ gas stripping, Absorption, Redox balance

## Abstract

Acetaldehyde is a platform chemical with a production volume of more than 1 Mt/a, but is chiefly synthesized from petrochemical feedstocks. We propose the fermentative conversion of glucose towards acetaldehyde via genetically modified *S. cerevisiae*. This allows for ethanol-free bioactaldehyde production. Exploiting the high volatility of the product, in situ gas stripping in an aerated reactor is inevitable and crucial due to the respiratory toxicity effects of the acetaldehyde overproduction. We devise a lab-scale setup for the recovery of the product from the off-gas. Water was chosen as a suitable solvent and the Henry coefficient of acetaldehyde in water was validated experimentally. Based on an experimentally verified capture efficiency of 75%, an acetaldehyde production rate of over 100 mg/g/h was reached in 200 mL lab-scale fermentations.

**Supplementary Information:**

The online version contains supplementary material available at 10.1007/s00449-022-02697-w.

## Introduction

The chemical industry needs to go green(er). This is necessary to reach the goal of climate neutrality within the next three decades as demanded by the European Union [1]. The chemical industry uses over 650 Mt/a of petrochemical feedstocks and, on top of that, over 510 Mt/a of hydrocarbons for process energy [2]. Besides costly Power-to-X approaches, biotechnological production of platform chemicals from renewable carbon sources (i.e. biomass, CO_2_, and waste) can reduce and at one point eliminate the use of fossil resources.

In industrial biotechnology, *Saccharomyces cerevisiae* is one of the most frequently used hosts, with the bioethanol sector as the prime example reaching 76.5 Mt/a in 2015 [3]. With nowadays easy genetic modifications, the feedstocks can range from glucose over lignocellulose biomass, H_2_/CO_2_ up to plastic monomers and crude oil [4, 5]. The range of products is almost limitless, ranging from alkenes, alcohols, organic acids over pyridines up to peptides, enzymes, and pharmaceutical proteins. However, the efficient purification of these products from the aqueous medium remains challenging and is the primary factor in manufacturing costs for the overwhelming majority of products [6].

Acetaldehyde is a large-volume commodity chemical, as it is a possible precursor for acetic acid, pyridines, as well as 1,3-butylene glycol. Further, it is used as a fine chemical in food and flavour industries. The global market consumption of acetaldehyde exceeds 1 Mt/a and is expected to grow in the next decade [7, 8]. The primary production route is through the Wacker-Hoechst process, the partial oxidation of ethylene, which in turn is mostly obtained from steam-cracked fossil resources [9]. A major field of research is the catalyst-driven dehydrogenation of bioethanol to acetaldehyde which enables acetaldehyde production based on renewable resources, hence bioacetaldehyde production [10–12]. For example, the Pörner Group offers this as a pre-planned industrial facility [13]. Although this process is already used in industrial scale, it can be enhanced in terms of efficiency, as acetaldehyde is an intermediate in bioethanol production, and 15% of the bioethanol production cost emerge in the downstream processing [14] A direct bioacetaldehyde production would circumvent the need for two purification steps.

Acetaldehyde as the precursor of ethanol in *S. cerevisiae* was discovered in the 1910s, among others by the work of Neuberg, although he targeted glycerol production. He used calcium sulfite to chemically bind the nascent acetaldehyde in yeast ferments in order to accumulate glycerol [15].

The next step for the compound’s overproduction was the establishment of *Zymomonas mobilis* as a production host by Wecker et al*.* in the 1980s, using the natively upregulated NADH oxidase activity in aerobic environments and thus limiting the reduction of acetaldehyde to ethanol. The volatile acetaldehyde is stripped from the fermenter and is bound by reacting with an aqueous sodium bisulfite solution in a subsequent trap. In this first targeted fermentation towards acetaldehyde, yields of 40% and a capture efficiency of 80% were achieved [16, 17]. Tanaka et al*.* optimized this method and further explored the combination of water traps and reactive precipitation via a 3-methyl-2-benzothiazolinone hydrazone hydrochloride solution in consecutive traps. This method gave a 94% overall recovery of acetaldehyde [18]. In Kalnenieks et al*.*, the expression levels of the NADH oxidase, as well as the alcohol dehydrogenase were changed through genetic modifications. This resulted in very high yields, but this study circumvented the recovery of acetaldehyde by measuring the concentration in the off-gas [19]. Other works focus on establishing new host organisms by introducing the *Z. mobilis* pyruvate decarboxylase gene into other microorganisms [20, 21].

All these approaches rely on limited acetaldehyde to ethanol conversion but cannot prevent ethanol production entirely. We propose for the first time bioacetaldehyde production without the co-production of ethanol. For that, we chose *S. cerevisiae* as the main host of the closely related ethanol production. Further, we strive to bypass the need for reactive compounds to bind acetaldehyde, as Wecker et al*.* and Tanaka et al*.* did. Hence, we follow a different approach by using water traps only and demonstrating the capture efficiency of traps in a lab-scale proof of concept. Rightfully, industrial volatile organic compound (VOC) emissions are heavily restricted [22]. In order to comply with these restrictions, the proposed system with water traps is not sufficient. To release the exhaust gas with the desired purity and under usage of reduced solvent demand, an absorption column in counter-current mode would be required [23]. The usage of an absorber column was already suggested for a large-scale process for the production of acetaldehyde from ethanol. Here, the acetaldehyde is produced in the gas phase and then absorbed to water. Consecutively the acetaldehyde is separated from water in a distillation step [24]. However, such a system is not applicable in lab-scale, which is why it is not further investigated in this study.

**Fig. 1:**
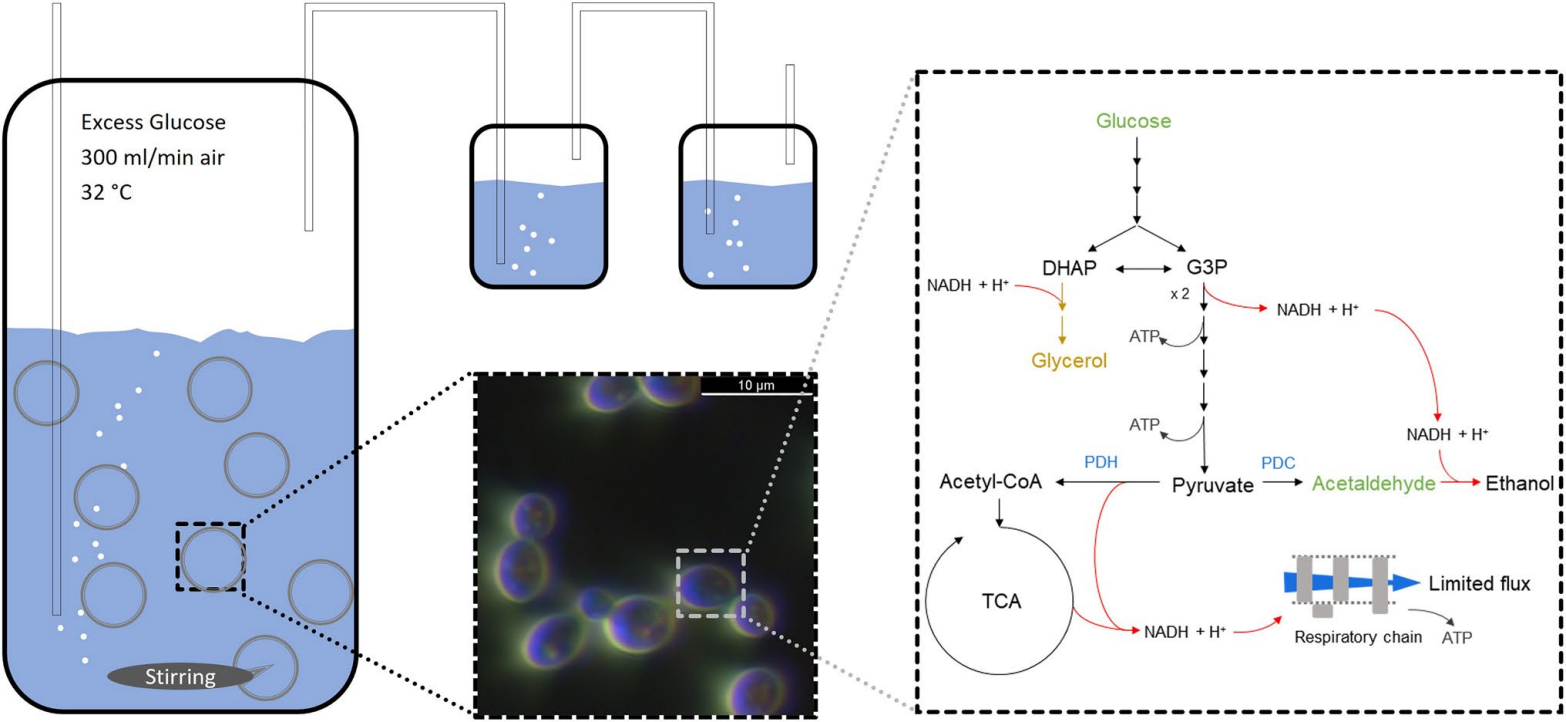
Production concept and network graph of acetaldehyde and generated ATP and NADH during glycolysis, the TCA, and ethanol production. In blue are the enzymes of the pyruvate branching: the pyruvate dehydrogenase (PDH, left) and the pyruvate decarboxylase (PDC, right)

To overproduce acetaldehyde without adding high amounts of reactive chemicals in yeasts, as Neuberg et al*.* did, the reaction towards ethanol needs to be eliminated [15]. This can be done by deleting all alcohol dehydrogenases (ADHs) and enzymes with ADH activities. This deprives the yeast of its ability to grow under anaerobic conditions, as the redox cofactor NADH cannot be regenerated through the formation of ethanol. Therefrom results the need to manage the carbon flux at the pyruvate branch (see Fig. 1). In aerobic environments, normally, all glycolysis products flow into the tricarboxylic acid cycle (TCA) and the electrons bound in NADH subsequently towards the respiratory chain. *S. cerevisiae* belongs to the Crabtree-positive yeasts. After exceeding a glucose threshold of only 0.1 g/L depending on the strain, respiration is down-regulated, and the excess pyruvate is transferred to the ethanol branch via the conversion to acetaldehyde [25, 26]. This work develops a novel fermentative production strategy of acetaldehyde based on renewable resources with genetically modified *S. cerevisiae*. The in situ stripping of acetaldehyde is not only inevitable in aerated fermenters but also favourable to reduce the toxic effect of acetaldehyde [27]. Obviously, stirring, aeration rates, and the fermentation temperature have a significant impact on the evaporation rates, but their optimization is out of scope for this work. In a consecutive step, we implement and investigate suitable separation techniques to capture the acetaldehyde from the gas stream to quantify the acetaldehyde.

## Materials and methods

### Used strain

The strain used is based on the *S. cerevisiae* CEN.PK113-17a including knockouts for all seven alcohol dehydrogenases and in genes for two multifunctional alcohol dehydrogenases, namely *SFA1* and *GRE2*. Thus the genotype is *MATα*; *ura3-52*; *leu2-3_112*; *TRP1*; *HIS3*; *MAL2-8C*; *SUC2*, *Δadh1, Δadh2, Δadh3, Δadh4, Δadh5, Δadh6, Δsfa1, Δgre2* [26]. This is a haploid laboratory strain chosen for the ease of using the leucine deficiency for CRISPR/Cas9-assisted genetic engineering.

### Yeast cultivation and media

Precultures were grown in liquid yeast extract peptone (YEP, for a more detailed composition see S1.1) medium at 30 °C in shaking flasks. Main cultures were grown in Verduyn minimal medium or optimized Verduyn minimal medium (V^+^, for a more detailed composition see S1.1) [28]. For the fermentations, a double-walled glass reactor with a working volume of up to 250 mL was used but only filled with 200 mL medium. The reactor was heated with a thermostat to 30 or 32 °C and stirred with the maximum possible speed of around 800 rpm with a triangular, 3.7 cm-long stirring bar. Compressed, humidified air was introduced via a needle with 0.8 mm diameter at 1.5 vvm (i.e. 300 mL/min) (for a more detailed setup see S1.2, Fig. S1.1, and Fig. 1). Higher aeration rates facilitate acetaldehyde evaporation but at the same time hinder acetaldehyde capture, therefore 1.5 vvm was chosen as upper limit of standard fermentation procedure [29].

The optical density at 600 nm was determined with a Ultrospec 10 (Amersham Biosciences, Little Chalfont, UK) photometer. Cell dry weight (CDW) was determined through a calibration curve following the growth of the strain in minimal media, yielding a correlation of 0.398 on OD/(g/L) CDW (for more detailed results see S2.1).

### GC and HPLC analytics

For GC-FID analytics, all samples were diluted in a ratio of 1:20 with acetonitrile (ACN) and directly stored in a screw-cap GC-vial at 4 °C. Samples that contained biomass were filtered through Berrytec CA 0.22 µm disposable syringe filters before dilution. Samples with high glucose content, e.g. uninoculated medium, tend to precipitate upon dilution; those samples were centrifuged for 2 min at 10,000 × g and decanted before storage in GC vials. A Trace GC Ultra GC-FID (Thermo Scientific, Waltham, MA, USA) was used with a 30 m Zebron ZB-WAX column with an inner diameter of 0.25 mm. The optimized sequence parameters are the following: a sample volume of 0.1 mL, a flow rate of 1 mL/min with helium as carrier gas and a split ratio of 1:10. The temperature program was as follows: 50 °C for 8 min, increased by 27.5 °C/min to 160 °C, and kept constant for 3 min. The inlet was kept at 250 °C.

For HPLC analytics, all samples were filtered through Berrytec CA 0.22 µm disposable syringe filters before analysis. A DIONEX UltiMate 3000 HPLC System (Thermo Scientific, Waltham, MA, USA) with a Metab-AAC column (300 × 7.8 mm column, ISERA, Düren, Germany) was used. Elution was performed with 5 mM H_2_SO_4_ at a flow rate of 0.4 mL/min and a temperature of 40 °C. For detection, a SHODEX RI-101 detector (Showa Denko Europe GmbH, München, Germany) and a DIONEX UltiMate 3000 Variable Wavelength Detector set to 210 nm were used.

### Determination of Henry coefficients

Property prediction for acetaldehyde and multiple solvents was performed by screening charges calculated by the conductor-like screening model (COSMO) using COSMOthermX19. COSMO for real solvation (COSMO-RS) was applied to gain activity coefficients by using the COSMOtherm internal BP-TZVPD-FINE parametrization, which refers to the Becke–Perdew functional and the triple zeta valence plus polarization function [30]. Based on the activity coefficients, the Henry coefficient of acetaldehyde in a solvent was determined at 25 °C.

For the experimental validation of the Henry coefficient, the dynamic equilibrium was determined by applying the gas stripping method as described in previous studies [31, 32]. Acetaldehyde was inserted into a Schott flask with three well-defined feeding rates of 0.8–2.1 mmol/L/h. The flask is flooded by an airstream with a flow rate of 300 mL/min, leading to a partial pressure of acetaldehyde in a range between 0.0011 and 0.0031 bar. The airstream was introduced to a water trap containing 250 mL of liquid. Samples with a volume of 0.5 mL were taken from the trap for 24 h as described above and were analysed in the GC (for a more detailed setup see S1.2, for more detailed results S2.5). Based on the defined amount of acetaldehyde in the entering gas stream and the measured concentration of acetaldehyde in the first trap when in a stationary state, equilibrium was assumed, and the Henry coefficient ${H}_{i}$ was determined as follows:

1 $${x}_{i}\cdot {H}_{i}={p}_{i}$$

2 $$\frac{{p}_{i}}{p}={y}_{i}=\frac{{n}_{i}}{{n}_{\mathrm{gas}}}$$

3 $${x}_{i}=\frac{{c}_{i}\cdot {V}_{\mathrm{liq}}}{{n}_{\mathrm{liq}}},$$

where the partial pressure ${p}_{i}$ was calculated as the quotient of the molar amount of acetaldehyde *n*_*i*_ and the total molar amount of the gas phase *n*_gas_ by applying the ideal gas model. The molar fraction ${x}_{i}$ in the liquid phase was calculated via the measured concentration *c*_*i*_, the molar amount of acetaldehyde *n*_liq_ and the liquid volume *V*_liq_ in the water trap.

## Results and discussion

With an engineered *S. cerevisiae* in hand that did not produce any ethanol, a first proof of concept, a fermentation at a 200 mL scale was conducted. The aeration was set to 1.5 vvm. With the expected stripping of acetaldehyde, a concept for the recovery from the gas stream was needed. Given the boiling point of acetaldehyde at 20.8 °C, the easiest solution seemed to be the condensation from the off-gas. A glass condenser at 0 °C with an inner surface of roughly 170 mm^2^ was used.

The strain grew in the first 24 h of the fermentation to a density of approximately 2.8 g/L CDW. Samples taken from the reactor, as well as from the condensed off-gas, were analysed in the GC-FID and did not include any ethanol, confirming the knockout of the alcohol dehydrogenases to be successful and no alternative enzyme activity existing. At the end of the fermentation, a total of 22 mL liquid was found in the cold trap with an acetaldehyde concentration of 8.8 mmol/L. No traces of other volatile components were found in the GC measurement. The estimated yield on glucose was in the order of 0.2% (for more detailed results see S2.2).

### Efficiency test for condensers

Due to the distinct smell of acetaldehyde in the off-gas, we hypothesised that the product was not condensed entirely. Thus, we devised an efficiency test. The capture efficiency can be determined by injecting a known amount of acetaldehyde into the system and measuring the acetaldehyde in the condensate and the reactor. Since the fermentation produces acetaldehyde over time, rather than injecting all acetaldehyde at once, a syringe pump was used to inject a constant feed over 16 h (4.7 g in 50 mL H_2_O with 3.1 mL/h). The capture efficiency is calculated as acetaldehyde found in the condensate divided by acetaldehyde injected minus the amount left in the reactor. From the injected 107.3 mmol, only 0.76 mmol were found in a total of 3.5 mL of condensate. The experiment showed an efficiency of approximately 0.7%, but multiple runs showed clearly diverging results (for more detailed results see S2.3).

The low amounts captured via the condenser can be explained by the high dilution of acetaldehyde in the gas stream. Based on the approximated capture efficiency, the partial pressure of acetaldehyde can be estimated to be 0.0005 bar by assuming ideal gas conditions. For the condensation of a component, the partial pressure needs to exceed its saturation pressure at the given temperature. When comparing the partial pressure of 0.0005 bar with the saturation pressure of 0.351 bar at 0 °C calculated based on Antoine parameters from literature [33], it is evident that the partial pressure of acetaldehyde is lower by three orders of magnitude than the saturation pressure. Despite these calculations, little amounts of acetaldehyde were found in the condenser trap. This can be attributed to the idealised estimation of the ideal gas model or to the high solubility of the acetaldehyde in the condensed water. Hence, these results demanded a new method for product recovery.

### Absorption

For the purification of polluted gas streams, absorption is a standard procedure. The absorption of acetaldehyde either as a toxic component from room air or as a fermentation product has been investigated in these studies [16, 34]. However, the acetaldehyde was bound to amino acids or sodium bisulphite, respectively, via reactive absorption and was not processed further. This approach leads to an additional process step in purification, as the reactively bound acetaldehyde needs to be released. The release of the bound acetaldehyde can be achieved by the addition of a base, which in turn produces waste salts. In this study, we focus on developing an absorption procedure, which enables us to capture the acetaldehyde from a diluted gas stream and obtain it as a product solution. Therefore, we absorbed the produced acetaldehyde into an aqueous medium and could, in theory, increase the concentration of the acetaldehyde in the binary mixture via consecutive distillation. With this approach, we are able to collect acetaldehyde to quantify the produced amount in the fermentation.

Due to its simple handling and non-toxicity, water is an absorption medium of favour in this proof of concept study. To assess the gas solubility in equilibrium of acetaldehyde in water and to compare it to traditional organic solvents, the Henry coefficient was determined using the conductor-like screening model for real solvations (COSMO-RS) [35]. A small Henry coefficient indicates that the equilibrium for a component is strongly on the side of the liquid phase. Another criterion for the absorbent choice was the vapour pressure of the solvents [23].

**Fig. 2:**
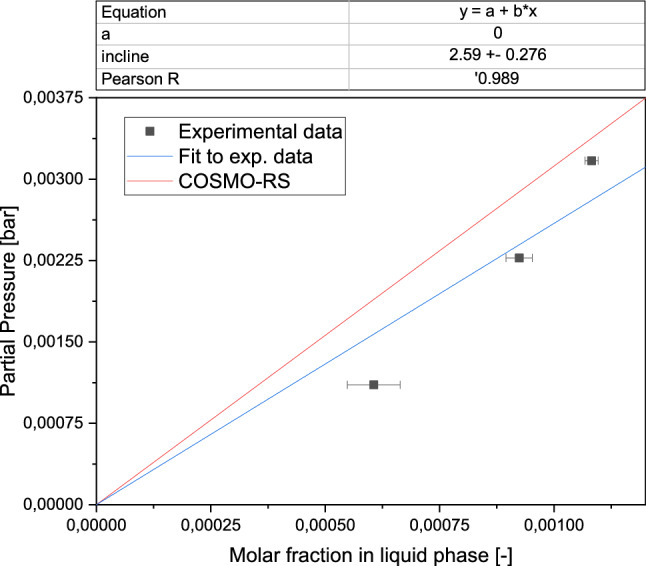
Correlation of molar fraction of acetaldehyde in the liquid phase and the partial pressure of acetaldehyde in the gas phase in bar at 25 °C. The slope of the black line refers to the predicted Henry coefficient using COSMO-RS calculations (3.098 bar). The experimentally derived measurement points are depicted as black squares. A linear regression line was calculated. The slope of the linear regression line represents the experimentally derived Henry coefficient which was determined to 2.592 bar. The experimentally determined Henry coefficient is in the same order of magnitude as the calculation based on COSMO-RS

Water was compared with eight organic solvents as an absorbent. Its relatively low predicted Henry coefficient (3.098 bar) and a sufficiently low vapour pressure of 0.0317 bar make it a reasonable choice of absorption medium (for more details see S2.4). Another advantage of water compared to the proposed organic solvents is that the water content in the off-gas has not to be considered as an additional component in further processing. The experimental determination of the Henry coefficient is based on the known entering acetaldehyde stream and the measured acetaldehyde concentration in one water trap. When the concentration in the water does not change over time, the two phases are assumed to be in equilibrium (for more details see S1.2 and S2.5). In Fig. 2, the molar fraction of acetaldehyde in water (horizontal axis) was correlated with the partial pressure of acetaldehyde in the gas phase (vertical axis). Three measurement points are depicted, representing partial pressures of acetaldehyde between 0.0011 bar and 0.0032 bar. A regression line was elongated through the measurement points and the origin. The slope through the measurement points and the origin represents the corresponding Henry coefficient. Hence, the experimentally derived Henry coefficient was determined to be 2.592 bar. The Henry coefficient derived from COSMO-RS is larger (3.098 bar) than the experimentally determined one, representing a lower affinity of acetaldehyde towards the absorbent. The larger Henry coefficient was chosen to estimate the minimal amount of solvent for the design of the absorptive separation sequence in order to conduct the estimation to the safe site.

The necessary amount of water for capture efficiencies of more than 70% in batch mode increases rapidly (see S2.9). This is due to a doubled demand of water to halve the acetaldehyde content remaining in the gas phase. Hence, a capture efficiency of 70% was targeted and the minimum amount of water needed to achieve this goal was calculated. In order to dissolve 70% of the inserted acetaldehyde from a gas stream of 300 mL/min over 16 h in the absorbent, approximately 1570 mL of water are necessary, assuming that the system consisting of water and gas phase is in equilibrium. Hence, a setup consisting of multiple consecutive water traps with a total water volume of 2000 mL was realized for the following experiments. The quantity of water traps was chosen to measure acetaldehyde accumulation over time and to obtain information about the equilibrium by analysis of the individual traps.

### Efficiency tests in the water traps

Instead of the condenser with a subsequent flask for acetaldehyde recovery, four water traps with 250 mL each and a fifth with 1000 mL were implemented. To enlarge the specific surface area of the air bubbles and thus enhance mass transfer, a sparger consisting of a tube with 0.8 mm holes was used in every trap. For a stable 1.5 vvm airflow despite the heightened resistance, the pressure was increased up to 1.4 bar. Although pressure influences the Henry coefficient in theory, small pressure changes below 10 bar are neglectable [36]. To enhance the evaporation of acetaldehyde from the reactor, the fermentation temperature was increased to 32 °C, the upper optimum limit for *S. cerevisiae* [37]. Since water was chosen as solvent, there was no need to remove moisture from the off-gas (for a more detailed setup see S1.2, for more detailed results see S2.6).

**Fig. 3:**
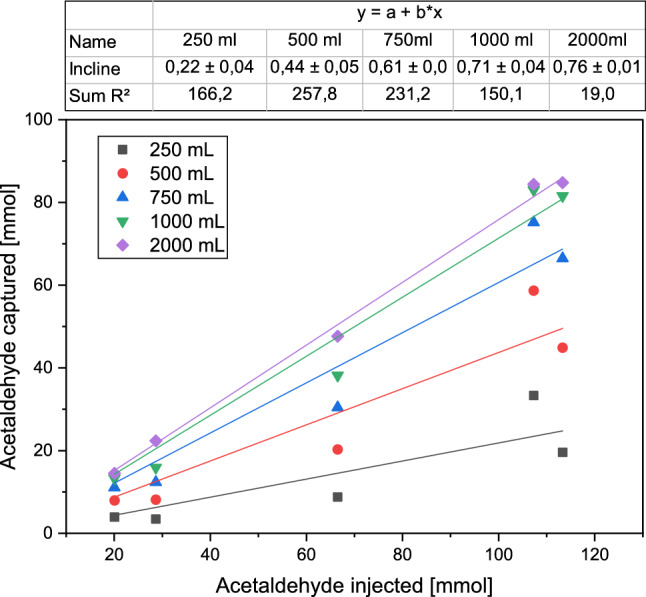
Acetaldehyde trapping efficiency tests. Cumulative capture efficiency of the water trap system consisting of five consecutive traps for different amounts of acetaldehyde fed after 24 h. The capture efficiency is calculated as acetaldehyde found divided by acetaldehyde injected minus the amount left in the reactor

Analogous to the condenser tests, acetaldehyde was fed to the aerated reactor and the capture efficiency was calculated based on consecutive GC analysis. In total, this system is able to capture 75 ± 3%, along all tested loadings of acetaldehyde between 20 and 120 mmol (see Fig. 3). With the determined constant capture efficiency of this setup, the amount of produced acetaldehyde in the following fermentations can be quantified.

### Fermentative production of bioacetaldehyde in lab-scale

The same strain was used again, but this time in an optimized medium with more trace elements, vitamins, and supplements (V^+^, for more details see S1.1). A fed-batch operation mode was implemented with an initial glucose concentration of 20 g/L at inoculation and a steady feed. The feed started after 3 h with 5 g/L/h for 16 h to ensure excess glucose conditions. To gain deeper insights into the cellular metabolism, two BlueSens BCP-CO_2_ sensors were implemented; one before the reactor and one after the first four water traps. The CO_2_ signal is the difference between the carbon dioxide concentrations in v/v % of inflow air and off-gas.

**Fig. 4:**
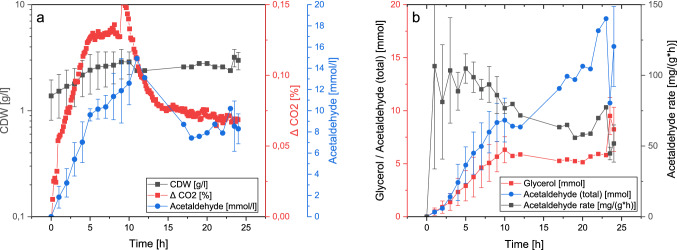
Fermentations with acetaldehyde capture. **a** CDW, CO_2_ signal (difference between the carbon dioxide concentrations in v/v % of inflow air and off-gas), and reactor supernatant acetaldehyde concentration as a mean with standard deviation (*n* = 3 for h 0–3,24; *n* = 2 for 4–10, 23 and *n* = 1 for 12–23, for CO_2_ *n* = 3 for h 0–9, *n* = 2 for 9–24). **b** Total amount of glycerol and acetaldehyde (reactor and traps) produced in the 200-ml setup, and rate of acetaldehyde production cumulated from the beginning of the fermentation as $({\mathrm{acetaldehyde}}_{tn}-{\mathrm{acetaldehyde}}_{t0})/({t}_{n}-{t}_{0})/CD{W}_{tn}$ *n* = 3 for h 0–3,24; *n* = 2 for 4–10, 23 and *n* = 1 for 12–23). For all individual figures see S2.7

Figure 4a shows biomass, CO_2_ signal, and acetaldehyde concentration in detail for one fermentation (for more detailed results see S2.7). The cells grew with a short lag phase and reached a stationary phase within the first 10 h of the fermentation. The CO_2_ signal increases after inoculation with the biomass, but declines after the cell reach the stationary phase. An increase in biomass beyond the peak cellular activity is most likely due to the usage of intracellular storage molecules.

But why is a steady-state reached at such low biomass concentrations? In all three fermentations, acetaldehyde accumulates in the reactor at concentrations of up to 10 to 15 mmol/L. A non-changing concentration of acetaldehyde in the reactor means an equilibrium of production rate and gas stripping. This concentration is described to inhibit glucose-dependent respiration in *S. cerevisiae* [38]. This fits our data, as reaching this level directly correlates with the peak of the CO_2_ signal. The mechanism behind the acetaldehyde intoxication is yet to be elucidated.. This means that the cells, even with the abundance of C-source and oxygen in the medium, begin to suffocate, as their ability for anaerobic growth is purposefully deleted. A declining CO_2_ production was observed in all three fermentations, whereby the stationary level of the biomass concentration varied with the inaccuracy in inoculation and the low maximum growth rate in the reactor. This is because, although the acetaldehyde production is primarily dependent on the biomass, the acetaldehyde concentration in the reactor in the first part of the fermentation is also time dependent, as the vessel is saturating. Even though a considerable amount of acetaldehyde is stripped with the gas stream, an accumulation to toxic levels was not prevented in this proof-of-concept setup.

The acetaldehyde in the off-gas stream is captured in a series of water traps. The amount of acetaldehyde produced over the course of the experiment is 12 mmol or 0.55 g on average (see Fig. 4b). And no traces of other components were found in the GC measurements. As already mentioned, we calculated the capture efficiency to be 75%. Considering the loss of 25% of product, up to 16 mmol or 0.71 g of acetaldehyde were produced (for the calculations see S2.6).

Taking a closer look at the dynamics of acetaldehyde production, the specific production rate is depicted in Fig. 4b (for a more detailed calculation see S2.7). The rate stays constant at around 100 mg/g/h in the first 6 h of the fermentation. After that the rate slows down to at least 50 mg/g/h. Because acetaldehyde derives from the primary metabolism, it is expected to be produced during all stages of the fermentation.

After glycolysis, the pyruvate could either flow towards acetaldehyde production or respiration (TCA-cycle). The distribution of C-atoms between these two options is dependent on the bottleneck respiratory capacity. A higher glycolytic rate and lower respiration capacity should increase the bottleneck and thus shunt more carbon into the production of acetaldehyde. Since it is assumed that acetaldehyde negatively influences the respiratory chain [27], an increased production rate is to be expected.

However, the experimental data shows a decline in the acetaldehyde production rate, of which possible reasons are shortly discussed here. For once, the metabolite could interfere with glucose uptake, glycolysis, or the pyruvate decarboxylases, but studies find only minor implications on the anaerobic CO_2_ production of *S. cerevisiae* strains stressed with acetaldehyde [38]. The second possibility is a simple end-product inhibition, as acetaldehyde has a low diffusion rate out of the cell and thus can accumulate multiple-fold compared to outside concentration [39]. To access this question, whether the observed concentration of 10 to 15 mmol/L acetaldehyde in the supernatant is indeed a limit, experiments without gas stripping were performed. The inoculated medium was cultivated in sealed Hungate-tubes with residual air for 24 h. This yielded titers of over 50 mmol/L acetaldehyde (for more detailed results see S2.8), not supporting the hypothesis of end-product inhibition. This leads to a third explanation: an impaired redox balance.

As seen in Fig. 1, sources of NADH are glycolysis, the pyruvate dehydrogenase, and the TCA cycle, while the major sink is the respiratory chain. As in Crabtree-positive yeasts, glycolysis outruns the respiratory capacity, reduction equivalents usually are additionally regenerated in the acetaldehyde to ethanol conversion [25, 28]. This is possible as ethanol (*γ*_*x*_ = 6.0) has a higher degree of reduction than glucose (*γ*_*x*_ = 4.0). Since the genes encoding the enzymes catalysing this reaction are deleted in the present strain, it must use alternative pathways to produce other reduced substances, such as glycerol (*γ*_*x*_ = 4.7). Here, one NAD^+^ is regenerated at the cost of one pyruvate that cannot be converted into biomass or acetaldehyde. The reason for the probable redox disequilibrium during the fermentation should be attributable to the two main products: biomass (*γ*_*x*_ = 4.17) and acetaldehyde (*γ*_*x*_ = 5.0). Thus, the glycerol concentration in the medium increases rapidly in the first 10 h of the fermentation, where biomass and acetaldehyde are produced (see Fig. 4b). At the point of arrested biomass production, the rate slows down, but glycerol is produced until the end of the fermentation, caused by continuous acetaldehyde production (see Fig. S2.6f. The interesting effects of the redox imbalance should be further investigated, for example, with the determination of intracellular metabolite and cofactor concentrations or metabolic flux analysis.

## Conclusion

The production of the platform chemical acetaldehyde using renewable materials by genetically modified *S. cerevisiae* is possible. We could, at rates between 50 and 100 mg/g/h produce on average 0.7 g acetaldehyde in 200 ml scale, which corresponds to a yield of 38%. In contrast to other publications [16–19], the presented strain does not form ethanol as a side product. We also integrate in situ gas stripping in the fermentation and consecutive absorption of the acetaldehyde. Water was chosen as a suitable solvent. The Henry coefficient for acetaldehyde in water was further determined experimentally, and the obtained value agreed with the predicted value. Based on this, a water trap system was designed, and a near constant capture efficiency of approximately 75% for a range of possibly produced amounts of acetaldehyde was observed. A balance in the reactor of production and removal was reached at a concentration of 10 to 15 mmol/L acetaldehyde. It is well known that acetaldehyde acts respiratory toxic, but the exact mechanism of aerobic acetaldehyde intoxication is still elusive. Due to the respiratory toxicity effect of acetaldehyde, a decrease in CO_2_ production and arrested growth in still excess glucose and oxygenic conditions was observed.

The high glycerol production, even at arrested growth, implies an impaired redox balance. The NADH produced during glycolysis cannot be regenerated in the conversion to ethanol, as this activity is absent in the engineered yeast. Regeneration by respiration is hindered when the acetaldehyde threshold in the cells is reached, as the compound is known to inhibit respiration. Glycerol is generally regarded as an unwanted side product. There are numerous approaches to prevent the formation, of which multiple could be applied for future acetaldehyde production studies [40].

In summary, we have performed a new approach of bio-acetaldehyde production within *S. cerevisiae*. We successfully used an alcohol dehydrogenase deficient strain in a new lab-scale setup to produce as well as capture acetaldehyde without the co-production of ethanol. Further, we gained new insights on the production dynamics and the challenges to overcome in the future.

## Supplementary Information

Below is the link to the electronic supplementary material.

Supplementary file1 (DOCX 2013 KB)
