# Supplementary material for: Yeast-based production and in situ purification of acetaldehyde
Source: Bioprocess Biosyst Eng. 2022 Feb 8;45(4):761–9. doi: 10.1007/s00449-022-02697-w (PMC8948146; doi:10.1007/s00449-022-02697-w)
Supplement: Supplementary file 1 — Supplementary file1 (DOCX 2013 KB) [file 449_2022_2697_MOESM1_ESM.docx]

# **Yeast-based production and *in situ* purification of acetaldehyde**

Hendrik G. Mengers^a^, William Graf von Westarp^b^, Daniela Brücker^a^, Andreas Jupke^b^, Lars M. Blank^a^

HGM https://orcid.org/0000-0002-5098-164X, WvW <https://orcid.org/0000-0002-6137-8942>, DB https://orcid.org/0000-0001-6154-6515, AJ <https://orcid.org/0000-0001-6551-5695>, LMB <https://orcid.org/0000-0003-0961-4976>

a. Institute of Applied Microbiology - iAMB, Aachener Biologie und Biotechnologie - ABBt, RWTH Aachen University, Aachen, Germany

b. Fluid Process Engineering (AVT.FVT), RWTH Aachen University, Aachen, Germany

**Corresponding author:** Lars M. Blank lars.blank@rwth-aachen.de

**Keywords:** Acetaldehyde, *S. cerevisiae*, *in situ* gas stripping, Absorption, Redox balance

# Supplementary Material and Methods

## Yeast cultivation

To access acetaldehyde production, the *S. cerevisiae* strain was cultivated in Verduyn minimal salt medium with excess glucose. Later fermentations also had three times vitamins, supplements, and trace elements (V^+^). To prevent medium acidification, the N-source was switched from ammonium sulphate to urea.

The medium contained if not specifically mentioned 20 g/L glucose, 10 g/L potassium hydrogen phthalate, 2.3 g/L urea, 3 g/L, KH_2_PO_4_, 0,5 g/L MgSO_4_ 7 H2O, as well as 50 mL/L 100x trace elements, 5 mL/L of 1000x vitamins. To counter the deficiencies in the Cen.PK113-17a based strain, following supplements were given in concentrations of 0.12 g/L: L-histidine, L-methionine, L-leucine, uracil. The vitamin solution contained 0.05 g/L D-biotin, 1 g/L calcium D pantothenate, 1 g/L nicotinic acid, 25 g/L myo-inositol, 1 g/L thiamine hydrochloride, 1 g/L pyridoxine hydrochloride and 0.2 g/L p-aminobenzoic acid. The trace element solution consisted of 1.5 g/L EDTA, 0.45 g/L ZnSO_4_·7 H_2_O, 0.1 g/L MnCl_2_·4 H_2_O, 0.03 g/L CoCl_2_·7 H_2_O, 0.03 g/L CuSO_4_·5 H_2_O, 0.04 g/L NaMoO_4_·2 H_2_O, 0.45 g/L CaCl_2_·2 H_2_O, 0.3 g/L FeSO_4_·7 H_2_O, 0.1 g/L H_3_BO_3_ and 0.01 g/L KI. The pH was adjusted to 5 before sterile filtration.

Precultures were performed in shaking flasks with YEP-medium containing 10 g/L yeast extract, 20 g/L peptone, 20 g/L glucose and 20 g/L agar. The flasks were inoculated with 10 µl of a frozen stock and cultivated at 30 °C for 72 h. Before inoculation, the cells were centrifuged for 3 min at 3500 x g and subsequently washed with V^+^ medium.

A picture of the setup is depicted in Figure S1.1. To humidify the air, it was guided through a gas wash bottle filled with 200 ml water, before entering the reactor.

## Setup for Efficiency tests and determination of Henry coefficients

For the efficiency tests, acetaldehyde was injected via a syringe pump with a constant feeding rate of 3.1 mL/h over 16 h into the reactor liquid phase. The acetaldehyde was weighed and diluted to 50 mL with bi-distilled water and drawn up into a syringe. The solution was introduced into the reactor via a cannula through the lower septum besides the cannula for gassing, simulating acetaldehyde production. The reactor was aerated with a flow rate of 300 mL/min pre-humidified air. The off-gas was led into the water trap system and samples were taken after 24h and analysed via GC.

For the determination of the Henry coefficients, a constant loading of the airstream was crucial. Therefore, the acetaldehyde solution was injected into empty 100 mL Schott-Flasks. The air was guided to the bottom of the flask with a plastic tube, and it was mixed with a magnetic stirrer. The flask was flooded by the airstream, resulting in a constant loading of the gas. The water traps were not changed during the experiment. Sampling was performed hourly between 21 and 26 h, as a constant loading of the trap was not to expected shortly after the start of the experiment. For sampling, 0.5 mL of the water trap were taken up with a syringe through the outlet.


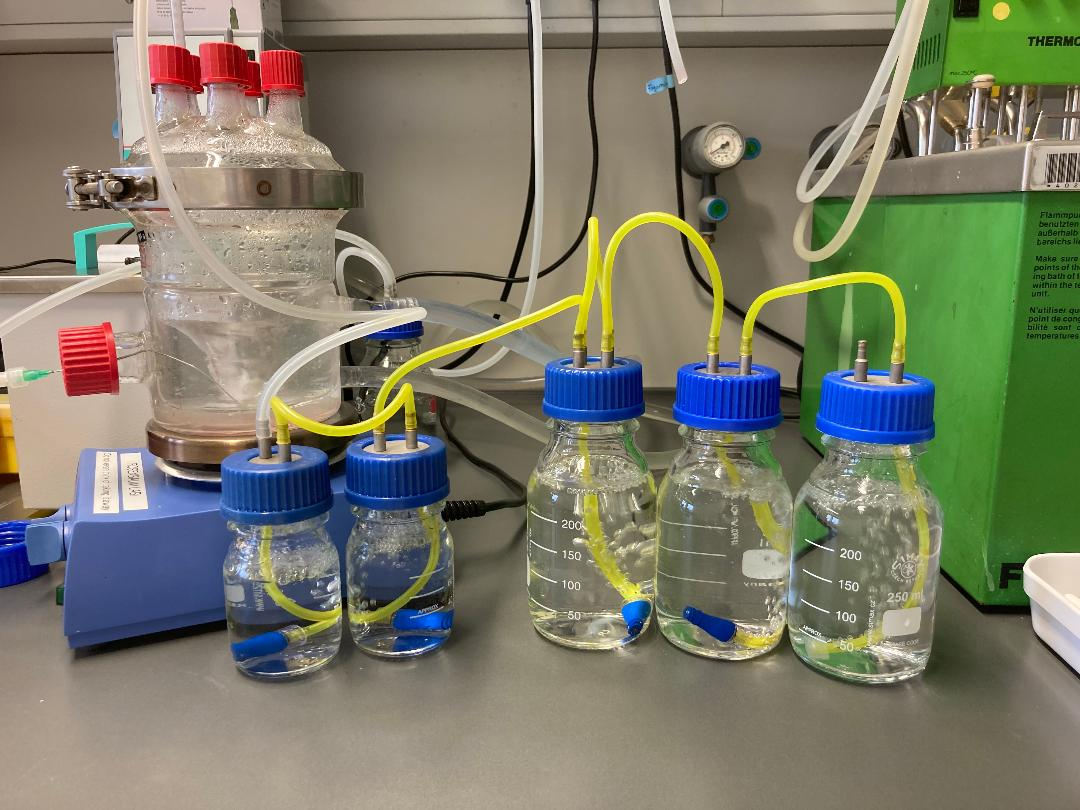

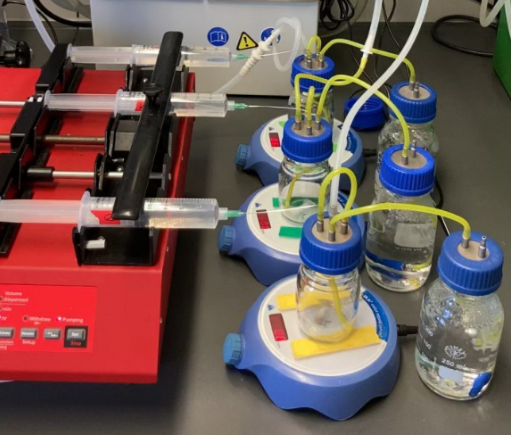


Figure 1.1 **a** Setup with the reactor and different size water traps and **b** for the determination of the Henry coefficients

# Supplementary Results

## Cell dry weight calibration

The cells were grown in V^+^ medium in shake flasks up to the desired optical density and then harvested. The cells were washed once with 0.5 x PBS buffer and subsequently dried and weighed. This yielded a correlation of 0.398 on OD/(g/L) CDW.

Figure 2.1 Calibration curve for OD to g/LCell dry weight calibration.

## Bioacetaldehyde fermentation with a condenser

In addition to what is described in the main paper, the strains were inoculated in Verduyn medium containing 20 g/L glucose monohydrate. Another 5 g glucose monohydrate in 10 ml demineralized sterile water were pulsed after the first depletion after 26.5 h. The glucose content was checked using MEDI-TEST Glucose/50 test-sticks from Macherey-Nagel. The setup is shown in Figure S2.2a and Figure S2.2bdepicts the biomass concentration over the curse of the fermentation. The fermentation temperature was set to 32 °C, the upper optimum limit of *S. cerevisiae* [37].

a
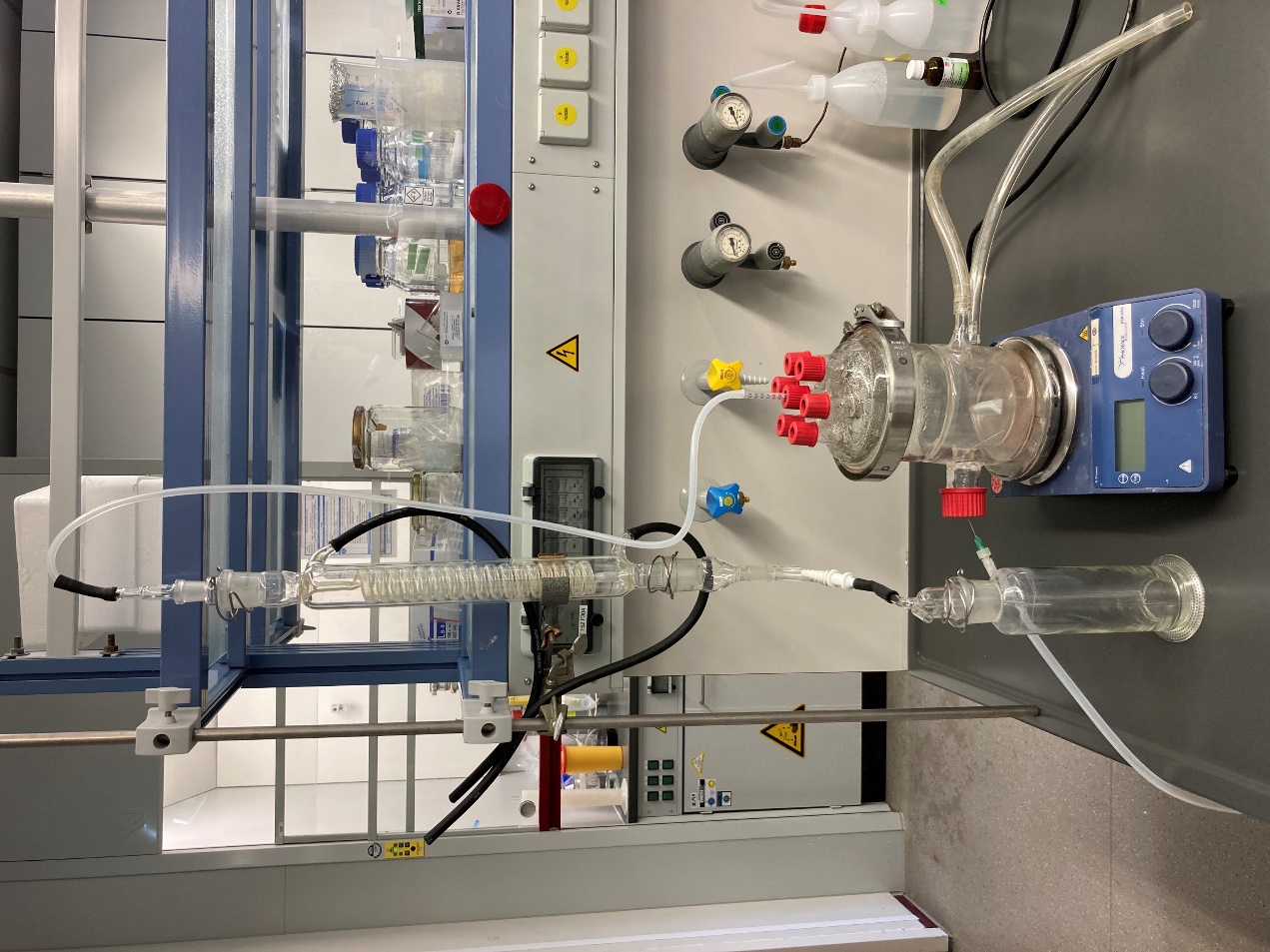
b

Figure 2.2 Bioacetaldehyde fermentation with a condenser. **a** setup of the fermentation, the collection vessel was cooled on ice **b** biomass concentration for the proof-of-concept fermentation.

To calculate the yield, the total volume of condensate had to be determined. As little droplets formed inside the condenser, it was necessary to flush it harshly with compressed air to force them out into the collection vessel. This was only feasible at the end of the fermentation since the condenser needed to be disconnected and moved strongly. Thus, there is no total amount and no yield for the 22.5 h measurement point. In addition to the poor capture efficiency, the handling was another disadvantage of the condenser setup.

At the end of the fermentation, no acetaldehyde was found inside the reactor. For the calculation of the yield, the theoretical optimum of 2 mol per mol glucose and the completely used 9 g glucose monohydrate were considered.

Table 2.1 Bioacetaldehyde fermentation with a condenser. Determination of volume and acetaldehyde content in the condensate. The yield is calculated from the theoretical maximum of 2 mol acetaldehyde per mol glucose.N.D. for not determined

| Time [h] | Acetaldehyde Concentration  [mmol/L] | Volume  of condensate  [L] | Acetaldehyde  [mmol] | Glucose consumed  [mmol] | Yield  [%] |
| --- | --- | --- | --- | --- | --- |
| 22.5 | 31.9 | N.D. | N.D. | 20.2 | N.D. |
| 75 | 8.8 | 0.022 | 0.193 | 45. 5 | 0.21 |

## Efficiency test with a condenser

In addition to what is described in the main paper, the acetaldehyde concentration in the reactor was monitored during the condenser efficiency test. Non-surprisingly, the acetaldehyde concentration in the reactor rises until the feed is empty and then depletes.

Figure 2.3: Acetaldehyde in the reactor (200 ml) throughout the first efficiency test with a total injection of 107.3 mmol

In addition to the test with the injection of 107.3 mmol, two further tests with lower loadings were performed. For the last one, no acetaldehyde was detected in the condensate and thus no efficiency can be calculated. We allocate the difference in volume found in the condenser mainly to varying amounts of water from the reaction.

Table 2.2 Acetaldehyde injected and found in the condenser after a 24-h experiment. N.D. for not determined.

|  | Injected  [mmol] | Volume  [mL] | Acetaldehyde  [mmol/L] | Acetaldehyde  [mmol] | Efficiency  % |
| --- | --- | --- | --- | --- | --- |
| Try 1 | 107.3 | 3.5 | 216.6 | 0.758 | 0.70 |
| Try 2 | 11.4 | 8 | 2.4 | 0.0192 | 0.17 |
| Try 3 | 11.3 | 5 | N.D. | N.D. | N.D. |

## Solvent screening

In addition to what is described in the main paper, a screening of possible solvents for the absorption of acetaldehyde was performed. The Henry coefficient, as well as the vapour pressure and the toxicity of the solvents, were evaluated. Even though the Henry coefficient of water is not as low as for some of the organic solvents, it was chosen due to its low vapour pressure and non-toxicity.

Table 2.3 Selection criteria for the choice of a suitable absorbent for acetaldehyde. The Henry coefficient was calculated using COSMO-RS at 1 bar and 25 °C. The vapour pressure was taken from literature at room temperature. The toxicity is evaluated based on the oral LD50 value for rats taken from the corresponding safety data sheets.

|  | Water | MTBE | 2-MTHF | CPME | Ethanol | Hexanol | Octanol | Octane | Decane |
| --- | --- | --- | --- | --- | --- | --- | --- | --- | --- |
| H_Acetaldehyde_  [bar] | 3.098 | 1.043 | 0.957 | 1.251 | 1.184 | 1.323 | 1.354 | 4.498 | 4.224 |
| P_D_  [hPa] | 31.69 | 270 | 129.7 | 59.9 | 59.5 | 1.23 | 0.11 | 18.8 | 1.91 |
| Toxicity  LD50 [mg/kg] | 90000 | 2000 | 300-2000 | 1000 | 10000 | 3210 | 5000 | 5000 | 5000 |

## Experimental determination of the Henry coefficient of acetaldehyde in water.

In addition to what is described in the main paper, the Henry coefficient was determined based on the experimental data. The setup is depicted in Figure S1.1b and the resulting acetaldehyde concentrations in the traps in Figure S2.4. The traps were sampled hourly after 21 h for 6 h. After 24 h the water trap is in equilibrium with the ingoing gas stream. Based on the acetaldehyde loading in the gas stream, which is known, and the acetaldehyde concentration in the water trap, which is measured, the Henry coefficient is calculated.


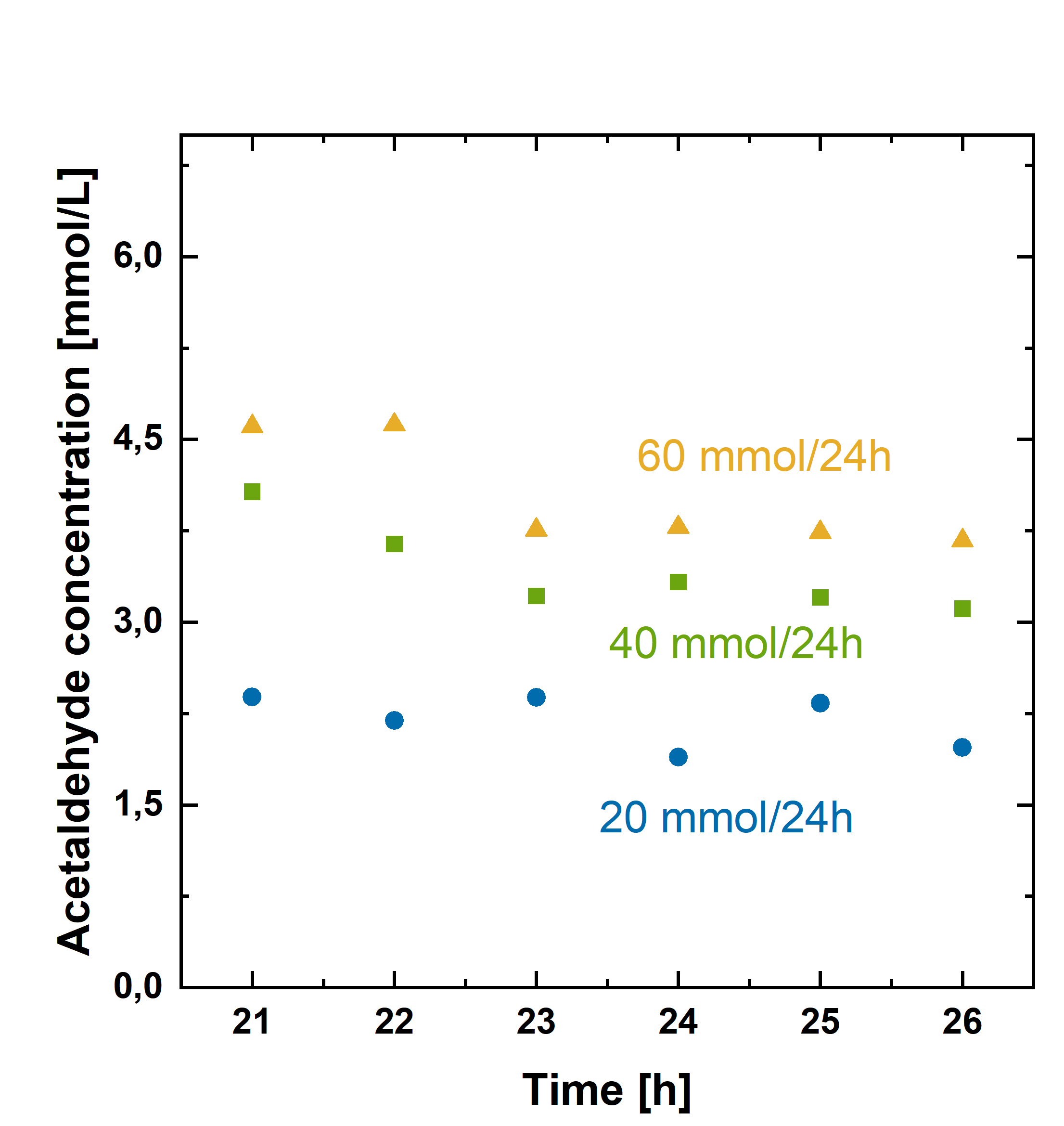


Figure 2.4: Acetaldehyde concentrations in the water trap with respect to time for different amounts of injected acetaldehyde into the gas stream over 24h.

## Efficiency test in the water traps.

In addition to what is described in the main paper, the acetaldehyde concentration in the reactor and the traps was monitored for the runs with 120 mmol acetaldehyde for multiple time points.

Figure 2.5: Acetaldehyde concentrations for the feeds with 114 mmol (h 1-5) and 107 mmol (h 16-24) in the medium and the five traps throughout the experiment.

As stated in the main paper, the capture efficiency is calculated to 75 ± 3 %. For the evaluation of acetaldehyde production in this system, the capture efficiency must be considered. But this obviously just applies to the acetaldehyde in the traps, not for the acetaldehyde measured in the medium inside the reactor. The calculation is as follows:

$$n_{ac}=\left( c_{ac_{reactor}}*V_{reactor} \right)+\left( \left( \frac{1}{0.75} \right)*\left( \left( c_{ac_{trap1}}*V_{trap1} \right)+ \ldots\right) \right)$$

Where *n_ac_* is the amount of acetaldehyde, *c_ac_* is the concentration of acetaldehyde in the respective reactor or trap, *V* is the volume of the respective reactor or trap.

## Fermentation in the complete system

In addition to what is described in the main paper, more data was collected. Figure 2.6 depicts biomass, CO_2_-signal (difference between the carbon dioxide concentrations in v/v % of inflow air and off-gas), and acetaldehyde concentration in the reactor for all three fermentations. Due to a minimal change in airflow, the CO_2_-signal of **a** shows a change, which is an artefact. The lower airflow also lowered the acetaldehyde evaporation rate, which can be seen as a jump in the concentration from 8.8 to 10.6 mmol/L in the reactor.

The acetaldehyde production rate is calculated with the total amount of acetaldehyde produced (reactor + traps) with capture efficiency considered. Since the production rate is mostly dependent on the number of cells and not the concentration (a larger reactor with the same total biomass would produce similar amounts), the total amount of biomass is used in gram cell dry weight. The calculation is as follows, where rate_ac_ is the rate of acetaldehyde production:

$$rate_{ac}={total acetaldehyde \left[ mg \right]}/{{biomass [g]}/{fermentation time [h]}}$$

| a | b |
| --- | --- |
| c | d |
| e | f |

Figure 2.6 Biomass (CDW), CO_2_-signal (difference between the carbon dioxide concentrations in v/v % of inflow air and off-gas) and acetaldehyde concentration in the reactor for all three fermentations throughout the experiment (**a-c**). Due to a minimal change in airflow, the CO_2_-signal of **a** shows a change, which is an artefact. **d** Total acetaldehyde found in the traps and the reactor with considered capture efficiency. **e** Rate of acetaldehyde production cumulated from the beginning of the fermentation as $(Acetaldehyde_{tn}-Acetaldehyde_{t0})/(t_{n}-t_{0})/CDW_{tn}$ **f** Glycerol concentration in the medium throughout the fermentation

Further, the amounts of glucose, glycerol, acetate, and acetaldehyde in the medium, as well as in the water traps were measured. The capture efficiency of 75.0 % was not considered for tables 2.3 to 2.5.

Table 2.3: Important metabolites for the first fermentation. The reactor had a volume of 200 ml.

| Time | Glucose | Glycerol | Acetate | Acetaldehyde (medium) | Acetaldehyde (sum all traps) |
| --- | --- | --- | --- | --- | --- |
| [h] | [mmol] | [mmol] | [mmol] | [mmol] | [mmol] |
| 1 | 19.4 | 0.9 | 0.4 | 0.1 | 0.1 |
| 2 | 18.0 | 1.7 | 1.0 | 0.3 | 0.3 |
| 3 | 15.3 | 2.4 | 1.8 | 0.8 | 1.0 |
| 4 | 20.9 | 3.6 | 1.9 | 1.8 | 2.4 |
| 5 | 25.1 | 4.3 | 1.9 | 2.0 | 3.4 |
| 6 | 30.7 | 5.6 | 1.6 | 1.8 | 4.6 |
| 7 | 35.4 | 6.5 | 1.9 | 1.9 | 5.2 |
| 8 | 40.3 | 6.8 | 1.9 | 1.8 | 6.1 |
| 9 | 47.4 | 7.1 | 1.4 | 1.8 | 6.7 |
| 10 | 53.0 | 7.5 | 1.7 | 2.1 | 6.8 |
| 23 | 83.5 | 11.2 | 2.2 | 2.2 | 12.4 |
| 24 | 84.1 | 11.0 | 1.8 | 1.8 | 12.4 |

Table 2.4: Important metabolites for the second fermentation. The reactor had a volume of 200 ml.

| Time | Glucose | Glycerol | Acetate | Acetaldehyde (medium) | Acetaldehyde (sum all traps) |
| --- | --- | --- | --- | --- | --- |
| [h] | [mmol] | [mmol] | [mmol] | [mmol] | [mmol] |
| 0 | 19.6 | 0.4 | 0.3 | 0.6 | 0.0 |
| 1 | 18.7 | 0.8 | 0.7 | 0.9 | 0.1 |
| 2 | 18.0 | 1.2 | 0.5 | 1.4 | 0.4 |
| 3 | 69.2 | 5.3 | 0.9 | 1.7 | 7.4 |
| 17 | 72.1 | 5.4 | 1.0 | 1.4 | 8.4 |
| 18 | 77.0 | 5.2 | 0.6 | 1.4 | 8.2 |
| 19 | 79.7 | 5.1 | 1.0 | 1.5 | 9.1 |
| 20 | 79.5 | 5.7 | 1.2 | 1.7 | 9.4 |
| 21 | 79.6 | 5.9 | 0.7 | 1.6 | 12.0 |
| 22 | 79.5 | 5.8 | 0.9 | 2.0 | 12.5 |
| 23 | 78.0 | 5.9 | 1.6 | 1.9 | 12.9 |
| 24 | 69.4 | 10.4 | 2.5 | 2.0 | 17.4 |

Table 2.5: Important metabolites for the third fermentation. The reactor had a volume of 200 ml.

| Time | Glucose | Glycerol | Acetate | Acetaldehyde (medium) | Acetaldehyde (sum all traps) |
| --- | --- | --- | --- | --- | --- |
| [h] | [mmol] | [mmol] | [mmol] | [mmol] | [mmol] |
| 1 | 20.4 | 0.1 | 0.0 | 0.3 | 0.0 |
| 2 | 20.1 | 0.3 | 0.3 | 0.7 | 0.0 |
| 3 | 19.7 | 0.5 | 0.4 | 0.9 | 0.4 |
| 4 | 21.8 | 1.0 | 0.5 | 1.0 | 0.4 |
| 5 | 27.7 | 1.5 | 0.5 | 1.8 | 1.0 |
| 6 | 30.4 | 1.9 | 0.7 | 2.2 | 1.3 |
| 7 | 34.4 | 2.7 | 0.7 | 2.3 | 1.6 |
| 8 | 37.5 | 3.3 | 0.7 | 2.7 | 2.5 |
| 9 | 39.6 | 4.2 | 0.9 | 3.0 | 3.1 |
| 10 | 42.9 | 5.1 | 0.8 | 2.9 | 3.1 |
| 11 | 46.5 | 5.7 | 0.8 | 3.0 | 4.2 |
| 12 | 50.2 | 5.9 | 0.8 | 2.6 | 4.4 |
| 23.5 | 78.7 | 7.8 | 1.3 | 1.2 | 6.8 |
| 24 | 77.9 | 7.8 | 1.2 | 1.2 | 6.8 |

## Cultivation in sealed Hungate-tubes

V^+^ medium with 20 g/L glucose monohydrate was inoculated to an OD of 1.6. The Hungate-tubes had a total volume of 16 ml and were filled with 4 ml of medium, thus 12 ml of air remained. The tubes were sealed and incubated at 30 °C for 24 h at 300 rpm. After that, the tubes were opened to relieve the pressure and the samples were taken as described above.

Unfortunately, as acetaldehyde is volatile, it is necessary to seal the reaction tubes. The 12 ml of air offers approximately only 25 mmol O_2_ per litre medium. The idealised assumption for acetaldehyde production is 1 glucose to 2 acetaldehyde. Further, 2 ATP and 2 NADH are produced. For the regeneration of the reduction equivalents, 1 O_2_ is needed. In theory, the production of 50 mM acetaldehyde could be possible with the amount of oxygen, but we assume that a considerable proportion of the oxygen is used otherwise

Table 2.6 Important metabolites for the cultivation in sealed Hungate tubes

| Duplicate | ΔCDW | Used Glucose | Acetaldehyde | Glycerol | Acetate |
| --- | --- | --- | --- | --- | --- |
|  | [g/L] | [mmol/L] | [mmol/L] | [mmol/L] | [mmol/L] |
| A | 2.2 | 101 | 50,4 | 78,3 | 12,3 |
| B | 1.8 | 101 | 42,3 | 82,8 | 12,8 |

## Calculation of minimum water volume for desired capture efficiency

In addition to what is described in the main paper, the minimum water volume for a desired capture efficiency can be calculated using the Henry coefficient and applying the ideal gas law. The necessary trap volume over the capture efficiency is depicted in Figure S2.7.

Figure 2.7: The minimum trap volume to capture a desired capture efficiency.
